# Neuroligin-2-Dependent Adhesion Defines a Molecular Checkpoint for Inhibitory Synaptic Plasticity

**DOI:** 10.1523/JNEUROSCI.1746-25.2026

**Published:** 2026-03-09

**Authors:** Anna Lech, Grzegorz Wiera, Jerzy W. Mozrzymas

**Affiliations:** ^1^Department of Biophysics and Neuroscience, Wroclaw Medical University, Wroclaw 50-368, Poland; ^2^Lukasiewicz Research Network – PORT Polish Center for Technology Development, Wrocław 54-066, Poland

**Keywords:** GABA, gephyrin, iLTP, inhibitory plasticity, inhibitory synapse, neuroligin-2

## Abstract

Long-term regulation of inhibitory synaptic strength is crucial for maintaining excitation–inhibition (E/I) balance in cortical circuits. In this study, we identify neuroligin-2 (Nlgn2) as a critical mediator of inhibitory long-term potentiation (iLTP) in hippocampal CA1 pyramidal cells (PCs). Using neurolide-2, a synthetic dendrimeric peptide that selectively interferes with Nlgn2–neurexin binding, in combination with whole-cell recordings in mouse hippocampal slices, we show that this interaction is required to maintain NMDA-induced iLTP. Disruption of Nlgn2–neurexin interactions blocked gephyrin clustering during iLTP and prevented Nlgn2 recruitment to GABAergic synapses, without affecting baseline inhibitory transmission. Immunostaining revealed that NMDA-induced enlargement of synaptic Nlgn2 clusters occurred selectively in the CA1 stratum oriens and was abolished by neurolide-2. Temporally controlled peptide application revealed a brief, 10 min post-induction window during which Nlgn2–neurexin adhesion is required for iLTP consolidation, and later application had no effect. Optogenetic experiments further demonstrated that NMDA-induced iLTP at both somatostatin (SST) and parvalbumin (PV) inputs depends on Nlgn2. In a more physiological paradigm, high-frequency stimulation of excitatory inputs paired with postsynaptic CA1 PC depolarization triggered heterosynaptic iLTP selectively at SST → PC synapses, which was unaffected during induction but failed to consolidate when Nlgn2–neurexin interaction was blocked, whereas excitatory LTP and PV-mediated inhibition remained intact. These findings identify perisynaptic Nlgn2–neurexin adhesion as an activity-dependent mechanism supporting inhibitory plasticity depending on input identity and induction protocol. Disruption of this process may impair inhibitory circuit remodeling, contributing to E/I imbalance in neurodevelopmental and psychiatric disorders.

## Significance Statement

The brain remains flexible and learns by adjusting the strength of excitatory and inhibitory synapses. While excitatory plasticity is well characterized, the rules guiding the induction and consolidation of inhibitory plasticity are less clear. We found that neuroligin-2, an adhesion protein that organizes inhibitory synapse formation, is also essential for inhibitory plasticity in the hippocampus, a brain region important for memory. Moreover, interference with neuroligin-2-dependent adhesion can erase already developed inhibitory plasticity within a short time window after induction, without affecting simultaneous plastic changes at excitatory synapses. These results highlight the consolidation phase of inhibitory plasticity and identify a mechanism that, if disturbed, may contribute to epilepsy, autism, and schizophrenia, which are linked to neuroligin-2 dysfunction.

## Introduction

Synapse organizers, once considered mainly as factors for synapse development and maintenance, are now recognized as active molecular regulators of synaptic plasticity and learning. They influence how excitatory synapses adapt to changes in neuronal activity (Connor and Siddiqui, 2023). Compared with excitatory synapses, GABAergic synapses rely on distinct combinations of adhesion molecules that govern the formation and stabilization of input-specific inhibitory circuits (Ko et al., 2015). Whether these adhesion proteins also contribute to the activity-dependent plasticity of inhibitory synapses remains largely unresolved.

Neuroligin-2 (Nlgn2), which forms perisynaptic complexes with presynaptic neurexins, is central to the architecture of inhibitory synapses. These complexes are involved in inhibitory synapse formation by coordinating the postsynaptic assembly of key components, such as gephyrin, collybistin, and GABA_A_ receptors (Ali et al., 2020). Beyond its developmental role, Nlgn2 remains crucial in mature neural circuits for maintaining inhibitory transmission and preserving excitation–inhibition balance (Wang et al., 2025). Deletion of Nlgn2 selectively reduces inhibitory postsynaptic currents (IPSCs) from parvalbumin-positive (PV^+^) fast-spiking interneurons while sparing inputs from somatostatin-positive (SST^+^) cells in both the hippocampus (Poulopoulos et al., 2009; Jedlicka et al., 2011) and somatosensory cortex (Gibson et al., 2009). This input-selective phenotype reflects the synapse-specific contribution of Nlgn2 to synapse formation. However, because Nlgn2 is broadly expressed at nearly all inhibitory synapses onto pyramidal neurons (Ali et al., 2020), it is likely to have additional functions beyond perisomatic synaptogenesis, potentially influencing other inhibitory inputs (Horn and Nicoll, 2018). For instance, Nlgn2 as a part of the transsynaptic adhesion complex is well placed to influence the long-term plasticity of inhibitory synapses. However, this potential role has received little direct investigation.

Long-term GABAergic plasticity is a fundamental mechanism by which inhibitory synapses adapt to changes in neuronal activity (Lourenço et al., 2010; Chiu et al., 2018; Field et al., 2020; Brzdąk et al., 2023; Wiera et al., 2024), with consequences for both circuit functions (Udakis et al., 2020) and cognition (Barron et al., 2017; Koolschijn et al., 2019). A key mechanism of inhibitory plasticity is the activity-dependent regulation of GABA_A_ receptors at the postsynaptic site, including their trafficking, clustering, and nanoscale organization (Petrini et al., 2014; De Luca et al., 2017; Pennacchietti et al., 2017). Moreover, different interneuron types engage distinct forms of GABAergic plasticity (Udakis et al., 2020; Wu et al., 2022a; Wiera et al., 2024), suggesting that the underlying mechanisms are synapse specific. This raises the possibility that intercellular adhesion, such as that between neuroligin-2 and neurexins, may contribute differently to plastic changes across inhibitory inputs.

Beyond intracellular mechanisms, growing evidence points to the extracellular environment, particularly the extracellular matrix (ECM) and its associated receptors in orchestrating inhibitory plasticity and its interaction with plastic changes at excitatory synapses. For example, the dystroglycan complex mediates GABAergic homeostatic scaling (Pribiag et al., 2014), and integrins, together with scaffolding proteins such as gephyrin, form signaling hubs that integrate ECM cues to modulate inhibitory strength (Wiera et al., 2022). Similarly, perineuronal nets and their proteolytic remodeling by enzymes such as MMP3 (matrix metalloproteinase 3) influence synapse-specific forms of inhibitory plasticity (Wiera et al., 2021, 2024; Jabłońska et al., 2024). These findings support the idea that cell-ECM adhesion and ECM reorganization are key to long-term inhibitory plasticity, raising the question of how perisynaptic cell–cell adhesion molecules integrate into this extracellular regulatory network.

To address this, we tested the hypothesis that adhesion between Nlgn2 and neurexins is involved in inhibitory synaptic plasticity. Using a synthetic peptide that competitively disrupts Nlgn2–neurexin interaction, we examined its effect on synapse-specific inhibitory long-term potentiation (iLTP) in CA1 pyramidal neurons. We found that blocking Nlgn2–neurexin binding abolishes iLTP and prevents the associated increase in postsynaptic gephyrin clustering. These results identify Nlgn2–neurexin adhesion as a critical mechanism underlying the expression of inhibitory synaptic potentiation. Altogether, our findings advance the understanding of inhibitory plasticity at synapse-specific molecular level and suggest possible links to the mechanisms of disorders, including schizophrenia, epilepsy, and autism.

## Materials and Methods

### Ethical approval and animals

All animal procedures in this study followed the ethical guidelines established by Polish law, specifically the Act on the Protection of Animals Used for Scientific or Educational Purposes (Act of January 15, 2015, with subsequent amendments) and conformed to EU Directive 2010/63/EU with amendments. Experiments involving genetically modified organisms were approved by the Polish Ministry of the Environment (approval numbers 144/2018 and 69/2023).

Brain slices were prepared from wild-type C57BL/6J mice of either sex bred at the Animal Facility of Wroclaw Medical University (Poland). To enable targeted optogenetic stimulation of defined inhibitory circuits, we also used several transgenic mouse lines obtained from Jackson Laboratory: Sst-IRES-Cre (stock #013,044), Pvalb-IRES-Cre (stock #017,320), and Ai32 (B6.Cg-Gt(ROSA)26Sortm32(CAG-COP4*H134R/EYFP)Hze/J; stock #024,109). Experimental animals were mainly double-transgenic SST-Cre::Ai32 and PV-Cre::Ai32 mice, which express channelrhodopsin-2 (ChR2) selectively in somatostatin- or parvalbumin-expressing interneurons, respectively. This approach allowed for the selective optogenetic activation of SST and PV inputs in pyramidal cells. All mice were housed in standard cages under a 12 h light/dark cycle, with food and water available *ad libitum*. Male and female mice were used in approximately equal proportions.

### Preparation of acute brain slices

Acute transverse hippocampal slices (350 µm thick) were prepared using two protocols, depending on the animal age. For older mice [postnatal days (P) 40–120], a protective recovery method optimized for neuronal viability was used based on the protocol described by Ting et al. (2018) and previously used in our studies (Jabłońska et al., 2024; Wiera et al., 2024). These slices were used in all experiments involving optogenetic stimulation (Figs. 5, 6). Mice were deeply anesthetized with isoflurane and rapidly decapitated. The brain was quickly removed and immersed in ice-cold, oxygenated (95% O_2_, 5% CO_2_) holding artificial cerebrospinal fluid (aCSF). The solution contained the following (in mM): 92 NaCl, 2.5 KCl, 1.2 NaH_2_PO_4_, 30 NaHCO_3_, 20 HEPES, 25 glucose, 5 sodium ascorbate, 2 thiourea, 3 sodium pyruvate, 1.3 MgSO_4_, and 2.5 CaCl_2_. The pH was adjusted to 7.4 using NaOH. After hemisecting the brain, each hemisphere was mounted on a vibratome (Leica VT1200 S), and transverse hippocampal slices were cut in the same ice-cold holding aCSF. Immediately after sectioning, slices were transferred to a 34°C bath containing an oxygenated recovery solution based on *N*-methyl-d-glucamine (NMDG), composed of the following (in mM): 93 NMDG, 2.5 KCl, 1.2 NaH_2_PO_4_, 30 NaHCO_3_, 20 HEPES, 25 glucose, 5 sodium ascorbate, 2 thiourea, 3 sodium pyruvate, 10 MgSO_4_, and 0.5 CaCl_2_, with pH adjusted to 7.4 using HCl. To gradually restore sodium levels and minimize osmotic stress, increasing volumes of 2 M NaCl (ranging from 0.25 to 2 ml) were added to the recovery solution (250 ml) over a 20 min period. This step is essential for preserving membrane integrity and improving gigaseal formation during patch-clamp recordings. Following recovery, slices were transferred back into the original holding aCSF and kept at room temperature for at least 1 h before use. Recordings were performed within 7 h of slice preparation.

**Figure 1.**
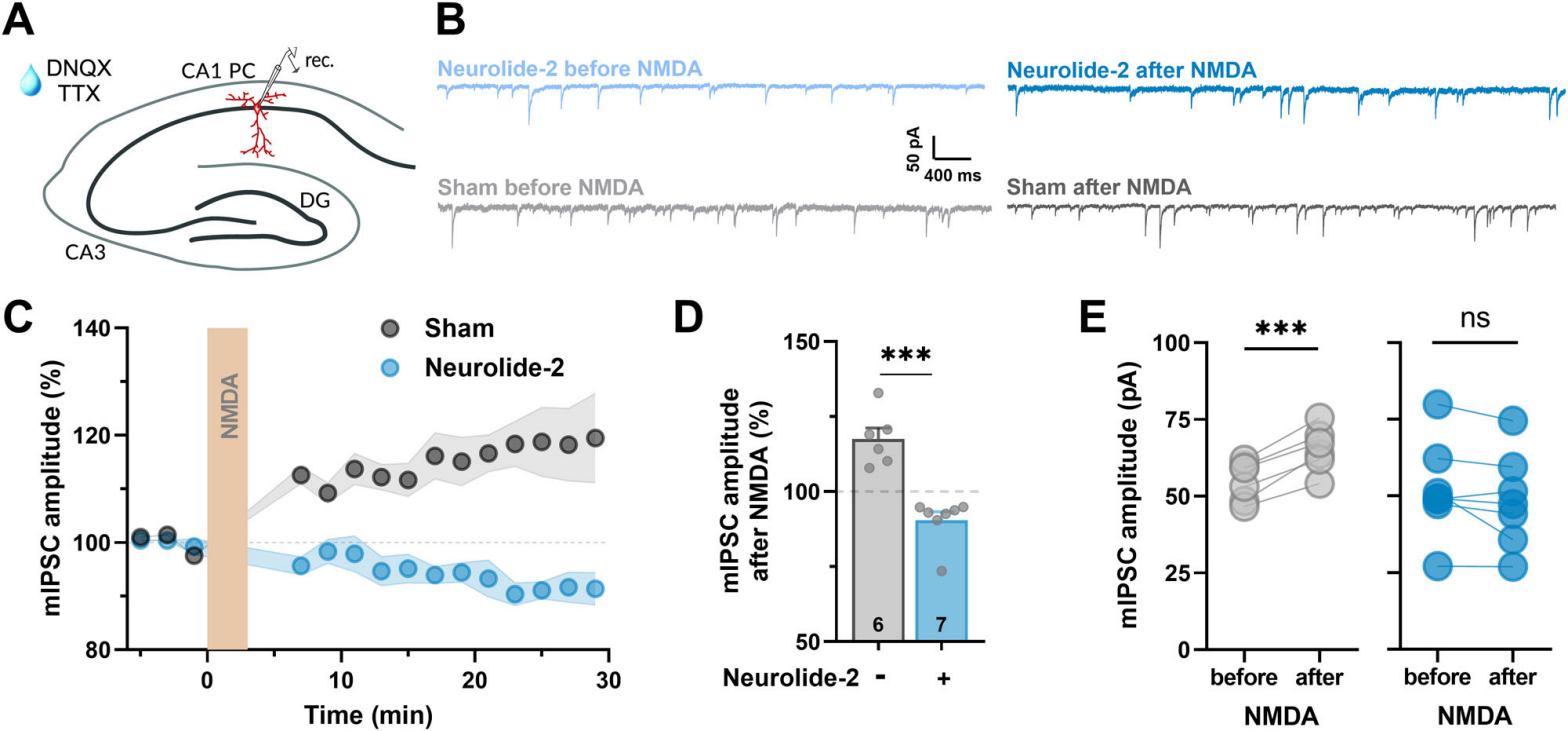
Neurolide-2 blocks NMDA-induced inhibitory synaptic plasticity in CA1 pyramidal neurons. ***A***, Schematic representation of whole-cell patch-clamp recordings from CA1 pyramidal neurons in acute hippocampal slices. Slices were incubated with TTX and DNQX to isolate GABAergic mIPSCs. ***B***, Representative traces of mIPSCs before and after NMDA application in the sham and neurolide-2-treated (blue) slices. ***C***, Time course of normalized mIPSC amplitude before and after NMDA (20 µM) application for 3 min (orange bar) in the sham and neurolide-2 (50 µM) conditions. Neurolide-2 shaded areas indicate SEM. ***D***, Summary of NMDA-induced changes in mIPSC amplitude, expressed as percentage of baseline and measured 20–24 min after NMDA application. Neurons exposed to neurolide-2 showed lower amplitudes than sham-treated cells (neurolide-2: 90.5 ± 3.7%, *n* = 6; sham: 117.5 ± 2.9%, *n* = 6). The dataset was evaluated with a one-way ANOVA that also included a neurolide-2-only group presented in Figure S1 (*F*_(2,18)_ = 20.6, *p* = 0.00002). Tukey's post hoc test confirmed a significant difference in NMDA-driven plasticity between the sham and neurolide-2 groups (adjusted *p* = 0.000022). ***E***, Paired analysis of mIPSC amplitudes before and after NMDA confirms iLTP induction in sham (before: 54.9 ± 2.6 pA, after: 65.4 ± 3.0 pA, paired *t* test *t*_(5)_ = 8.1, ***p = 0.00047), but no significant change following neurolide-2 treatment (before: 52.5 ± 6.1 pA, after: 48.5 ± 5.9 pA, *t*_(6)_ = 1.75, *p* = 0.13; ns, not significant).

**Figure 2.**
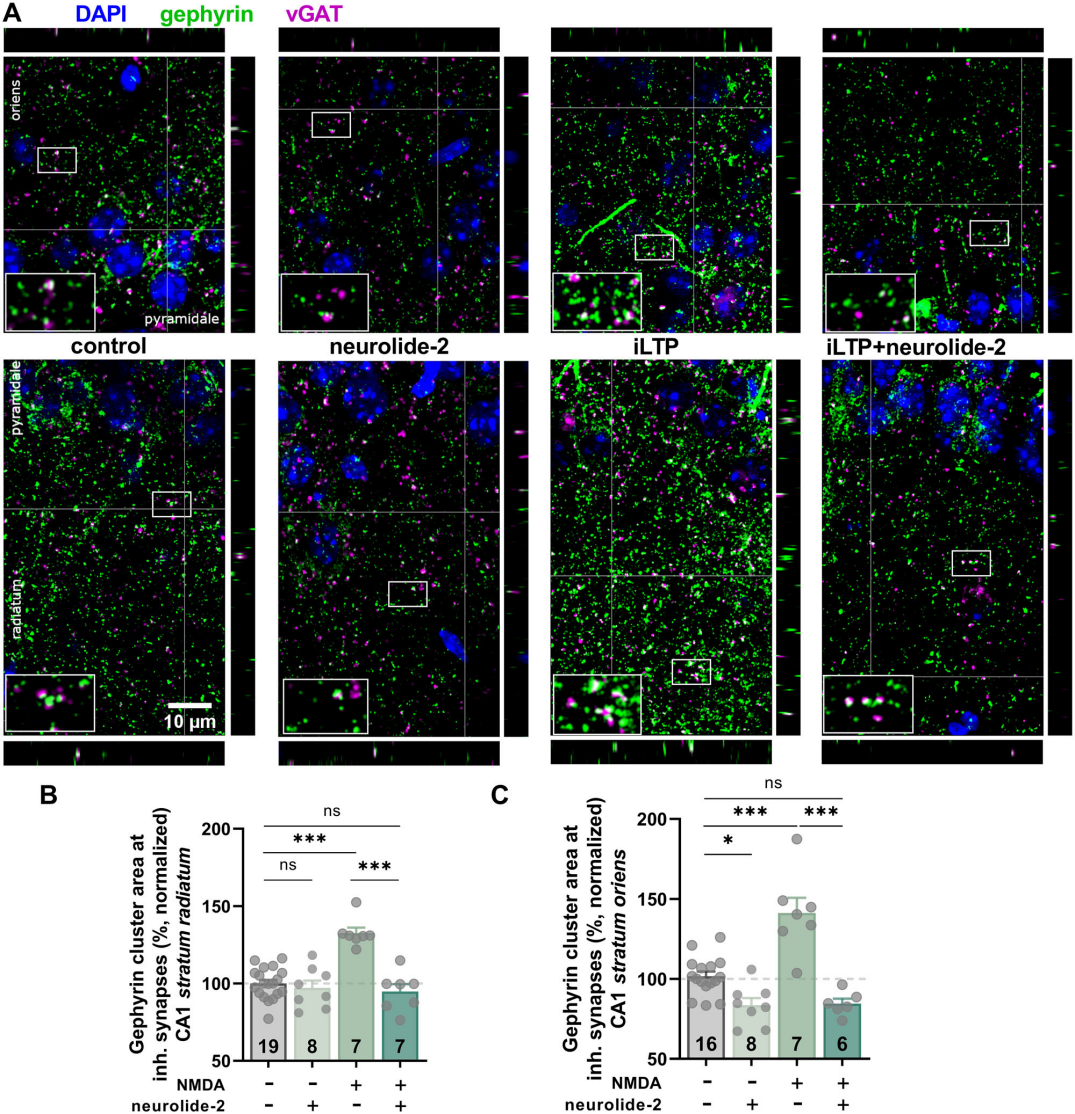
Neurolide-2 blocks NMDA-induced gephyrin accumulation at inhibitory synapses. ***A***, Representative confocal images showing gephyrin (green), vGAT (magenta), and DAPI (blue) immunostaining in the CA1 stratum radiatum (bottom) and stratum oriens (top) from four experimental groups: control and NMDA-treated slices (iLTP), each incubated with either scrambled control peptide or neurolide-2. Insets show enlarged views of gephyrin and vGAT colocalization (white), and small panels next to the main images show orthogonal views. ***B***, Quantification of the synaptic gephyrin cluster area (normalized to the control) in the CA1 stratum radiatum. iLTP induction with NMDA significantly increased the area of synaptic gephyrin clusters compared with sham controls (*p* = 0.0000006), an effect that was abolished by neurolide-2 (*p* = 0.000001 vs NMDA only). Slices treated with neurolide-2 alone did not differ from sham (*p* = 0.93); analyzed using one-way ANOVA with post hoc Tukey's test (control: 100 ± 2.3%, *n* = 19; neurolide-2:97.2 ± 4.6%, *n* = 8; iLTP: 132.6 ± 3.6%, *n* = 7; iLTP with neurolide-2:94.8 ± 4.8, *n* = 7; *F*_(3,37)_ = 19.2, *p* = 0.0000001). ***C***, Same analysis of CA1 stratum oriens. One-way ANOVA revealed a significant effect of treatment on synaptic gephyrin cluster area in the stratum oriens (*F*_(3,33)_ = 22.8, *p* = 3 × 10^−7^). Post hoc Dunnett's multiple-comparisons test identified a robust increase in the gephyrin cluster area following NMDA-induced iLTP compared with sham controls (control: 100 ± 3.0%; iLTP: 141.2 ± 9.5%, *p* = 8 × 10^−5^). This structural enhancement was fully prevented by neurolide-2; slices exposed to both NMDA and neurolide-2 showed no significant difference from the control (*p* = 0.10) but differed markedly from the iLTP group (*p* = 5 × 10^−6^). Neurolide-2 alone slightly reduced gephyrin cluster area (83.6 ± 4.5%) relative to controls (*p* = 0.042) but did not differ from the iLTP + neurolide-2 group (84.6± 3.2%, *p* = 0.99). Error bars show mean ± SEM. Individual data points represent slices. ns, not significant; *p < 0.05; ***p < 0.001.

**Figure 3.**
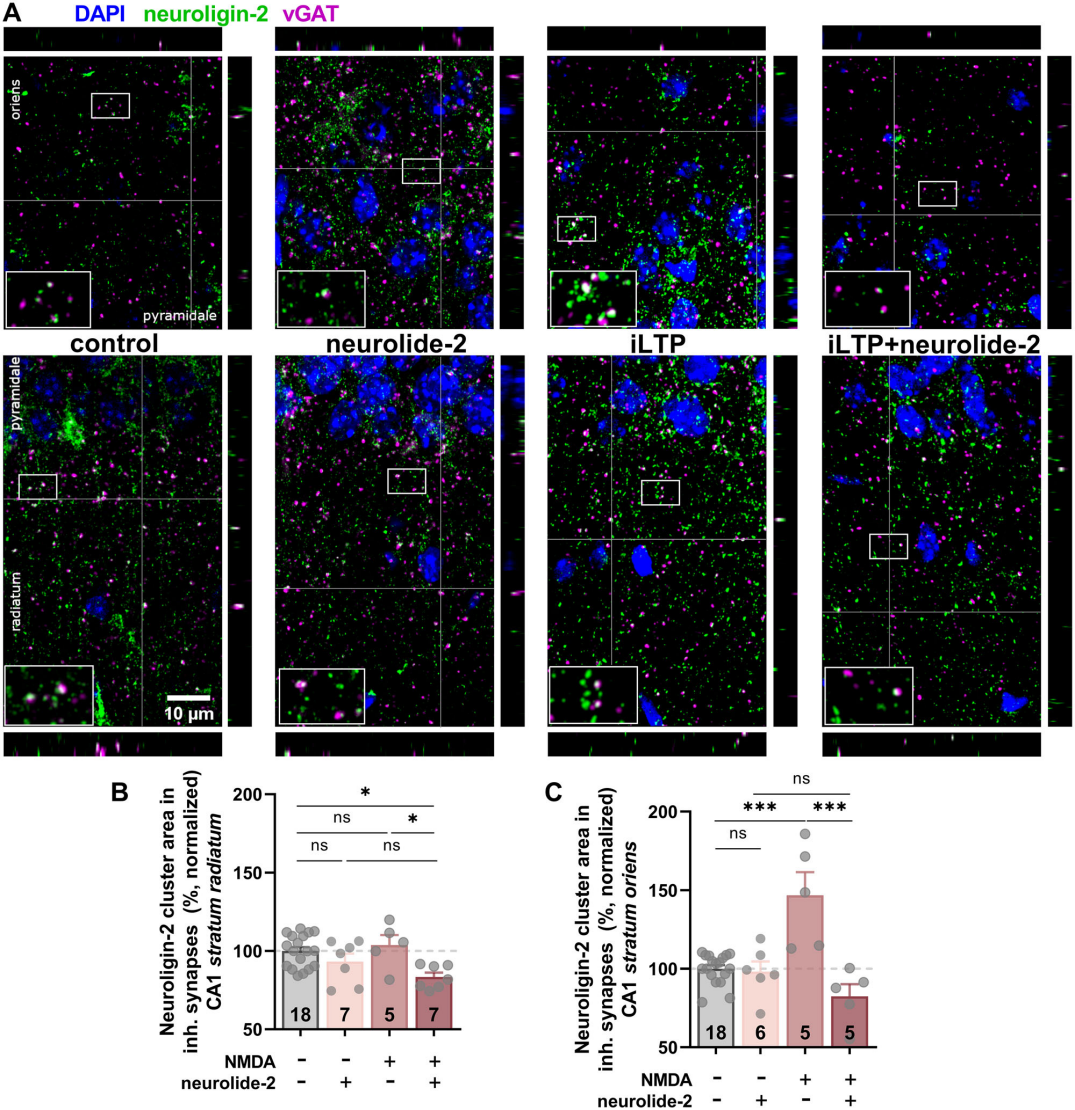
Neurolide-2 prevents NMDA-induced neuroligin-2 recruitment at vGAT-positive inhibitory synapses. ***A***, Representative confocal images showing neuroligin-2 (green), vGAT (magenta), and DAPI (blue) labeling in the CA1 stratum oriens (top row) and stratum radiatum (bottom row) in four experimental groups: control, neurolide-2 alone, NMDA-induced iLTP, and NMDA-iLTP in the continuous presence of neurolide-2. Insets display enlarged merged views highlighting neuroligin-2 and vGAT colocalization (white); small panels next to each main image show orthogonal projections. ***B***, Quantification of neuroligin-2 cluster area (expressed as % of control) at vGAT-positive inhibitory synapses in CA1 stratum radiatum. Neurolide-2 alone did not alter neuroligin-2 cluster size, whereas NMDA-iLTP combined with neurolide-2 significantly reduced the cluster area compared with iLTP alone (control: 100 ± 2.4%; neurolide-2:93.1 ± 5.2%; iLTP: 103.7 ± 6.4%; iLTP + neurolide-2:83.3 ± 2.8; *F*_(3,33)_ = 4.8, *p* = 0.0073; one-way ANOVA with post hoc Tukey’s test). ***C***, Same analysis of stratum oriens. Induction of NMDA-iLTP caused a marked increase in neuroligin-2 cluster size, which was prevented by neurolide-2 (control: 100 ± 2.2%; neurolide-2: 98.1 ± 6.6%; iLTP: 146.7 ± 14.7%; iLTP + neurolide-2: 82.5 ± 7.7; *F*_(3,30)_ = 14.4, *p* = 5 × 10^−6^; one-way ANOVA with post hoc Tukey's test). Error bars represent mean ± SEM; individual points indicate values from separate slices. ns, not significant; *p < 0.05; ***p < 0.001. Numbers on bars denote the number of slices analyzed from at least three animals per group.

**Figure 4.**
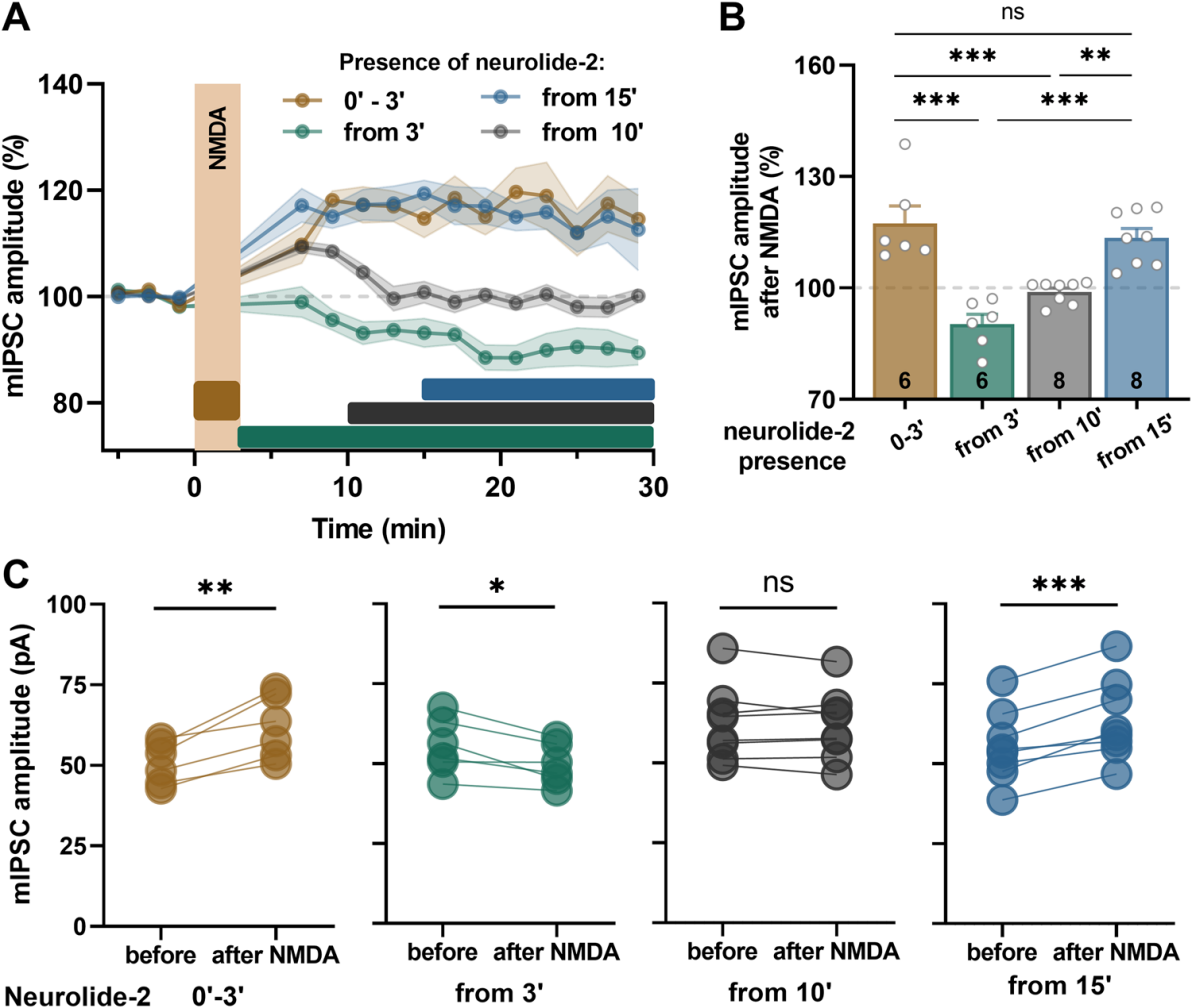
The timing of neurolide-2 application determines its impact on iLTP expression. ***A***, Time course of normalized mIPSC amplitudes in CA1 pyramidal neurons following NMDA-induced iLTP, with neurolide-2 applied during four different time windows: 0’–3’ (brown), 3’–30’ (green), 10’–30’ (gray), and 15’–30’ (blue). Shaded areas represent SEM; horizontal bars indicate neurolide-2 exposure duration; vertical bars represent NMDA application. ***B***, Summary of average mIPSC amplitude measured 20–24 min after NMDA in each group (0’–3’: 117.4 ± 4.7%; from 3’: 90.2 ± 2.6%; from 10’: 98.9 ± 1.1%; from 15’: 113.4 ± 2.6%; one-way ANOVA: *F*_(3,24)_ = 20.0, *p* = 2 × 10^−6^; Tukey's post hoc test: ***p < 0.001, **p < 0.01, ns: not significant. Each point represents a single cell; error bars indicate the mean ± SEM; numbers within bars indicate cell counts. ***C***, Paired comparisons of mIPSC amplitudes before and after NMDA administration in each condition. Neurolide-2 blocked iLTP only when applied during the 3’–30’ window (paired *t* tests; 0’–3’: *t*_(5)_ = 4.7, *p* = 0.0046, before 50.7 ± 2.6 pA, after 61.8 ± 4.0 pA; from 3’: *t*_(5)_ = 3.3, *p* = 0.021, before 55.5 ± 3.6 pA, after 49.8 ± 2.7 pA; from 10’: *t*_(7)_ = 0.55, *p* = 0.62, before 62.4 ± 4.2 pA, after 61.8 ± 3.9 pA; from 15’: *t*_(7)_ = 6.3, *p* = 0.0004, before 55.5 ± 4.0 pA, after 63.7 ± 4.5 pA). ***p < 0.001, **p < 0.01, *p < 0.05; ns, not significant.

For younger mice (P18–P25), a simplified procedure was used, similar to that described by Wiera et al. (2022). Following decapitation, brains were rapidly dissected and sectioned in ice-cold, carbogenated aCSF containing the following (in mM): 119 NaCl, 26.3 NaHCO_3_, 11 glucose, 2.5 KCl, 1 NaH_2_PO_4_, 1.3 MgSO_4_, and 2.5 CaCl_2_, adjusted to pH 7.4 and continuously saturated with 95% O₂ and 5% CO₂. After sectioning, hippocampal slices were transferred to a recovery chamber containing the same oxygenated aCSF and maintained at room temperature until use in mIPSC recordings and immunostaining (Figs. 1–4).

### Electrophysiological recordings

Whole-cell patch-clamp recordings were performed in acute hippocampal slices placed in a submerged recording chamber continuously perfused with oxygenated artificial cerebrospinal fluid at a flow rate of 4 ml/min (aCSF; 119 mM NaCl, 26.3 mM NaHCO_3_, 11 mM glucose, 2.5 mM KCl, 1 mM NaH_2_PO_4_, 1.3 mM MgSO_4_, and 2.5 mM CaCl_2_, pH 7.4). The temperature in the chamber was maintained at 28 ± 1°C. Neurons were visualized using an upright microscope (Zeiss Axio Examiner Z1) equipped with a 40× water immersion objective and infrared differential interference contrast optics.

Patch pipettes were pulled from borosilicate glass capillaries (GB150TF-8P, Science Products) using a P-10 micropipette puller (Sutter Instrument) to a final resistance of 3–5 MΩ (with below specified internal solution). For all optogenetic experiments, the pipettes were filled with an intracellular solution containing the following (in mM): 130 K-gluconate, 10 HEPES, 2 MgCl_2_, 2 MgATP, 0.3 NaGTP, 7 Na_2_-phosphocreatine, 0.6 EGTA, and 5 KCl, adjusted to pH 7.2 with KOH and osmolarity to 295 mOsm with K-gluconate. This solution contained a low physiological concentration of Cl^−^ to maintain hyperpolarizing IPSCs at the resting membrane potential. For mIPSC recordings, an internal solution with a high chloride concentration was used, which contained the following (in mM): 10 potassium gluconate, 125 KCl, 1 EGTA, 10 HEPES, 4 MgATP, and 5 sucrose, pH 7.25, 295 mOsm. The junction potential was not corrected.

Electrophysiological signals were recorded using a MultiClamp 700B amplifier, low-pass filtered at 4 kHz, and digitized at 20 kHz using a Digidata 1550A interface (Molecular Devices). Data acquisition was controlled using Clampex software (Molecular Devices). CA1 pyramidal cells were distinguished by their position in the stratum pyramidale and characteristic electrical properties, such as regular spike frequency adaptation and noticeable hyperpolarization-activated voltage sags. Throughout the recordings, the input resistance was consistently checked for stability, and cells showing fluctuations exceeding 20% of the baseline value were discarded from further analysis. After the initial assessment of firing patterns in the current-clamp mode, all subsequent recordings were conducted in the voltage-clamp configuration.

Miniature IPSCs (mIPSCs) were recorded in the presence of DNQX (20 μM) and tetrodotoxin (1 µM) at −70 mV membrane potential. To establish the reversal potential of optoevoked GABA_A_ receptor-mediated currents, inhibitory postsynaptic currents were measured at various holding potentials ranging from −90 to −55 mV. At synapses between PV-expressing interneurons and pyramidal cells, the average reversal potential of optogenetically evoked IPSCs was −72.8 ± 0.61 mV (*n* = 44).

### Optogenetic and electrode stimulation

**Figure 5.**
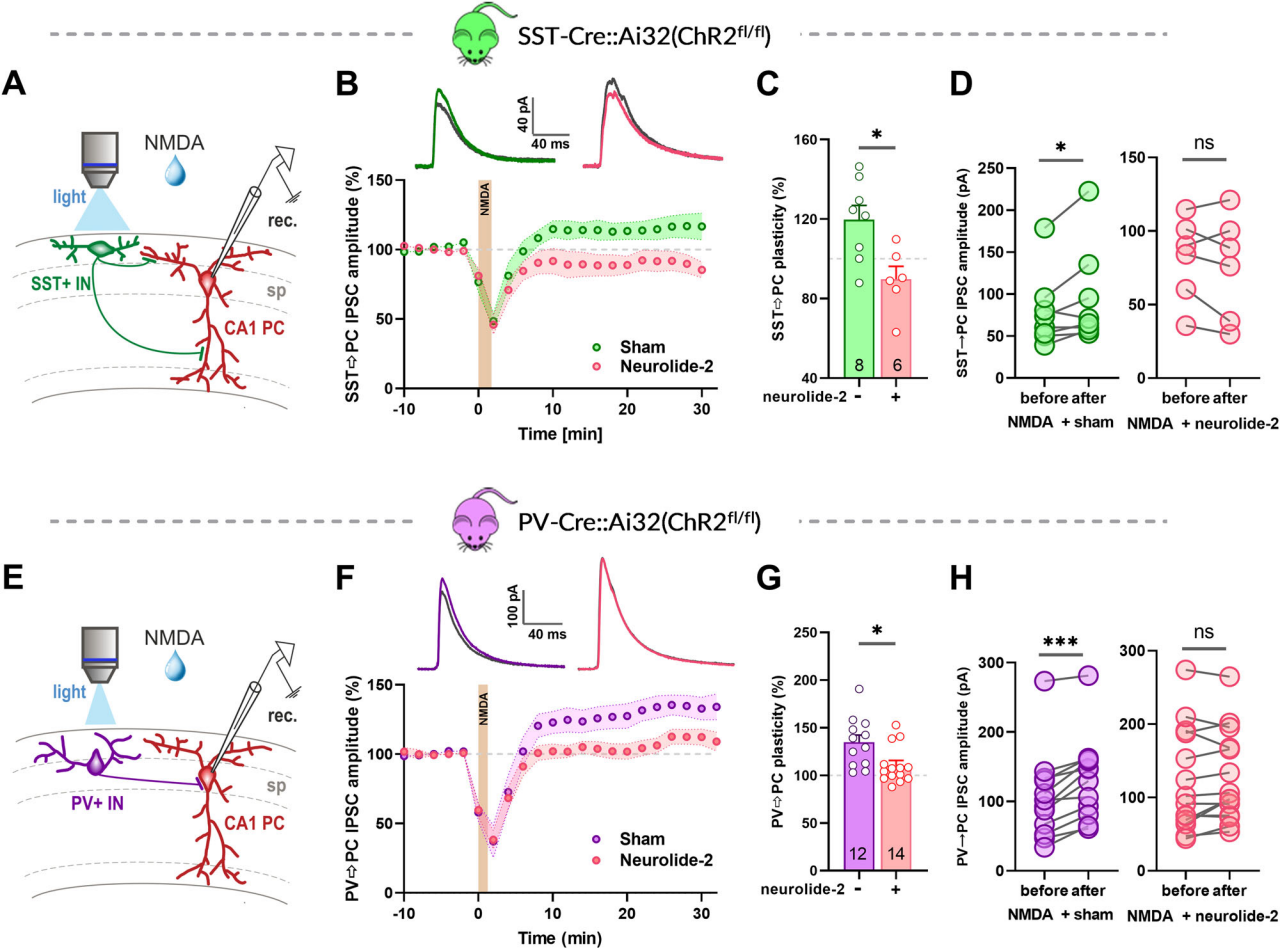
SST and PV interneuron synapses both rely on neuroligin-2 for NMDA-induced iLTP. ***A***, ***E***, Experimental design for local optogenetic activation of SST (***A***) or PV (***E***) interneurons expressing ChR2. NMDA (20 µM) was applied to induce iLTP of light-evoked IPSCs recorded in CA1 pyramidal neurons. ***B***, ***F***, Time course of the normalized IPSC amplitudes in the SST → PC (***B***) and PV → PC (***F***) pathways. The sham group treated with the scrambled peptide showed robust potentiation. Neurolide-2, bath applied throughout the recording, prevented iLTP induction. Shaded areas indicate SEM; orange areas mark NMDA application (1 min 45 s for SST → PC and 1 min 15 s for PV → PC synapses). ***C***, ***G***, Summary bar graphs of IPSC potentiation measured 26–30 min after NMDA administration (mean ± SEM). In both SST and PV pathways, neurolide-2 treatment significantly reduced iLTP compared with sham. A two-way ANOVA revealed a significant main effect of treatment (*F*_(1,36)_ = 14.3, *p* = 0.0006), with Sidak's post hoc tests showing adjusted *p* = 0.0277 for SST → PC and *p* = 0.013 for the PV → PC synapses. Numbers within bars indicate the number of recorded cells. ***D***, ***H***, Paired comparison of IPSC amplitudes before and after NMDA administration in the SST (***D***) and PV (***H***) groups. Sham-treated slices show iLTP (SST before: 79.0 ± 16.0 pA, after: 95.0 ± 20.6 pA, *p* = 0.039; PV before: 106.2 ± 18.3 pA, after: 132.7 ± 17.3 pA, *p* = 0.0005), whereas in the presence of neurolide-2 NMDA-iLTP is abolished (SST before: 81.0 ± 11.7 pA, after: 75.7 ± 14.5 pA, *p* = 0.44; PV before: 122.0 ± 18.8 pA, after: 127.4 ± 16.8 pA, *p* = 0.22). Nonparametric Wilcoxon test; ns, not significant; *p < 0.05; ***p < 0.001.

**Figure 6.**
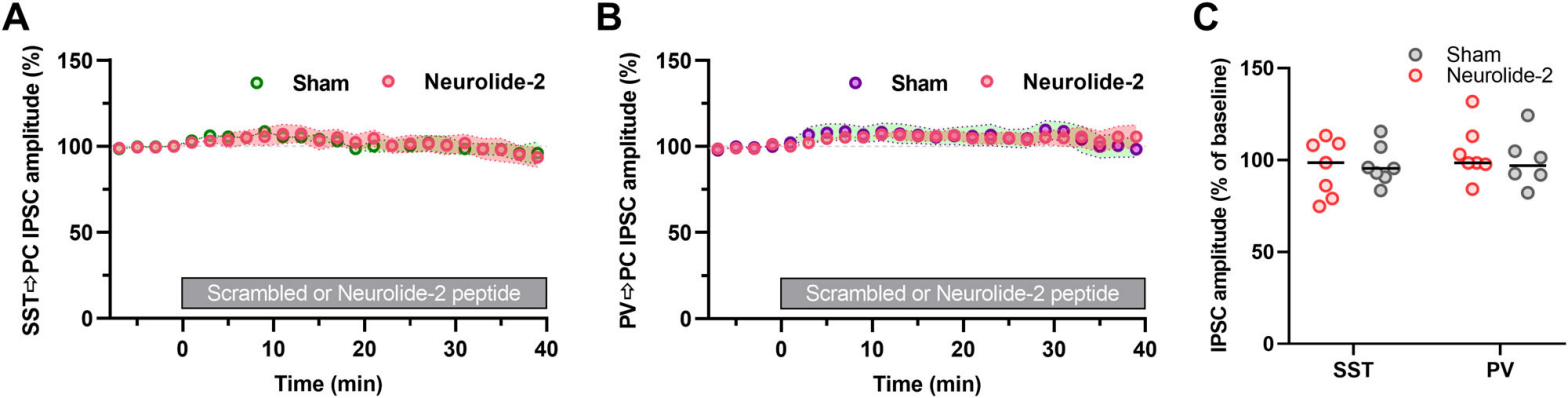
Neurolide-2 does not alter IPSC amplitudes in either SST or PV synapses onto CA1 pyramidal neurons. ***A***, ***B***, Time courses of normalized optogenetically evoked IPSCs in SST → PC (***A***) and PV → PC (***B***) inputs show stable amplitudes during 40 min of exposure to neurolide-2 or scrambled peptide. The gray bar indicates the period of peptide presence. ***C***, Comparison of changes in mean IPSC amplitude (relative to baseline) in 36–40 min after application of neurolide-2 or scrambled peptide shows no significant effect (SST → PC sham: 97.3 ± 4.0%, neurolide-2: 95.6 ± 5.9%; PV → PC sham: 99.6 ± 5.9%, neurolide-2: 103.8 ± 5.7%; two-way ANOVA *F*_(1,23)_ = 0.30, *p* = 0.59).

For experiments involving optogenetic stimulation (Figs. 5, 6), the source of blue light (DG-4, Sutter Instrument) was coupled to an optical fiber and directed onto the hippocampal slice through the microscope objective, passing through an HQ470/40× excitation filter (Chroma). Light pulses (fixed duration of 2 ms) were triggered via TTL signals delivered by the Digidata 1550A interface, allowing time-locked activation of channelrhodopsin-2 (ChR2)-expressing presynaptic interneurons. The light power density was ∼10 mW/mm^2^ at the tissue level. To selectively activate different interneuron populations, two illumination approaches were employed: (1) for SST → PC synapses, wide-field stimulation was used by fully opening the aperture of the objective, with the objective positioned over the stratum oriens to broadly activate SST-positive interneurons; (2) for PV → PC synapses, a restricted aperture (∼10% open) was used to focus light onto the pyramidal cell soma, minimizing off-target effects and ensuring selective activation of perisomatic PV terminals.

Postsynaptic pyramidal neurons were voltage-clamped at −60 mV to record optogenetically evoked IPSCs, allowing an adequate driving force for outward currents while maintaining physiological chloride concentrations. Concurrently, in some experiments (Fig. 6), Schaffer collaterals were stimulated using a stratum radiatum-positioned concentric bipolar electrode (FHC) to evoke CA3 → CA1 excitatory postsynaptic currents (EPSCs). EPSCs were recorded at −70 mV, near the reversal potential for IPSCs, to reduce the interference from inhibitory currents. Both synaptic responses were alternately triggered at 0.1 Hz with 5 s intervals between optogenetic and electrode stimuli. For excitatory stimulation, the current (200 μs duration) was adjusted to produce EPSC amplitudes of ∼40% of the maximum response without inducing action potentials.

To evoke GABAergic short-term synaptic plasticity, we applied paired-pulse stimulation with interstimulus intervals of 50 ms, 200 ms, 500 ms, 2 s, and 10 s. The paired-pulse ratio (PPR) was derived by dividing the peak amplitude of the second IPSC by that of the first.

### Long-term plasticity induction protocols

To induce NMDA-dependent inhibitory long-term potentiation (iLTP), we applied a widely used chemical protocol in which brain slices were exposed to 20 μM NMDA in the perfusion solution. The duration of NMDA application was adjusted depending on the experiment: 3 min for mIPSC recordings and immunofluorescence staining, 1 min and 15 s for PV → PC synapses, and 1 min and 45 s for SST → PC synapses, based on our previous studies (Wiera et al., 2022, 2024). In a subset of experiments, LTP at excitatory CA3 → CA1 synapses was induced using high-frequency stimulation (HFS) of the Schaffer collaterals. This protocol consisted of three 1 s trains at 100 Hz, delivered with 20 s intervals between each train. During HFS trains, the postsynaptic CA1 pyramidal neurons were held at 0 mV.

### Electrophysiological data processing methods

All recorded electrophysiological signals were processed using Clampfit 10.7 software. When analyzing mIPSCs, we manually identified individual events using a template. For each recording, we measured both the mIPSC amplitude and frequency over continuous 2 min periods. To quantify changes in synaptic strength following NMDA-induced iLTP, we divided the average mIPSC amplitude during the post-induction period (20–22 min after NMDA application) by the preinduction baseline average.

To examine the kinetics of optogenetically evoked IPSCs, we focused on two key features. First, we analyzed the onset kinetics by measuring the rise time from 10 to 90% of IPSC peak amplitude. Second, we analyzed the kinetics of the IPSC decay phase by fitting it with a double exponential function [*y*(*t*) = *A*_1_exp(−*t*/*τ*_fast_) + *A*_2_exp(−*t*/*τ*_slow_), where *τ*_fast_ and *τ*_slow_ represent the time constants for the fast and slow decay phases, respectively, and *A*_1_ and *A*_2_ are the corresponding amplitudes] and calculated the weighted average decay time constant that accounted for two components proportionally based on their respective amplitudes [*τ*_mean_ = *a*_1_⋅*τ*_fast_ + *a*_2_⋅*τ*_slow_, where *a*_1_ = *A*_1_/(*A*_1_ + *A*_2_) and *a*_2_ = *A*_2_/(*A*_1_ + *A*_2_)].

### Immunohistochemistry

Twenty-five minutes after NMDA-iLTP induction, hippocampal slices (350 µm thick) were fixed overnight at 4°C in a solution containing 2% paraformaldehyde and 0.2% picric acid in phosphate-buffered saline (PBS). The following day, slices were rinsed in PBS (three times, 15 min each), embedded in 15% gelatin (VWR Chemicals), and sectioned into 40 µm slices using a vibratome (Leica VT1000S). The sections were transferred to the wells of a 96-well plate and washed again in PBS (three times, 10 min each). The tissue was then incubated for 1 h at room temperature in a blocking solution containing 10% normal horse serum (NHS; Vector Laboratories) in PBST (PBS supplemented with 0.1% Tween-20, Sigma-Aldrich). Primary antibodies were applied in PBST containing 4% NHS and incubated for 48 h at 4°C on a shaker. The following primary antibodies were used: mouse anti-gephyrin (Synaptic Systems, catalog #147 111; 1:800), rabbit anti-vesicular GABA transporter (vGAT; Synaptic Systems, catalog #131 002; 1:500), mouse anti-neuroligin-2 (Synaptic Systems, catalog #129 511; 1:200), and mouse anti-active form of integrin β1 (Chemicon, catalog #MAB2079Z; 1:250). Following primary antibody incubation, sections were washed in PBS (three times, 10 min each) and incubated with the appropriate secondary antibodies for 24 h at 4°C with gentle shaking. The following secondary antibodies were used: Alexa Fluor 488 anti-mouse (Thermo Fisher Scientific, catalog #A-11029) and Alexa Fluor 633 anti-rabbit (Thermo Fisher Scientific, #A-21071), both diluted 1:1000 in PBS containing 4% NHS. The next day, slices were washed again with PBS (three times for 10 min each) and mounted on microscope slides using Fluoroshield mounting medium with DAPI (Sigma-Aldrich). Samples were stored at 4°C until confocal imaging.

### Image acquisition and analysis

Samples were imaged using an Olympus FluoView 1000 laser-scanning confocal microscope (Olympus). Immunofluorescence signals were acquired using a 60× PlanApo oil-immersion objective (numerical aperture 1.35). Images were collected in the sequential scanning mode to minimize spectral overlap. For each field of view, a *z*-stack of six optical sections spaced 0.95 µm apart was captured. On average, two regions of interest per slice were selected based on the hippocampal cytoarchitecture, encompassing contiguous portions of the stratum oriens, stratum pyramidale, and stratum radiatum within the CA1 region.

Image analysis was performed using the ImageJ software. For quantification, maximum intensity projections of the *Z*-stacks were generated, unless otherwise stated. The analysis focused on the CA1 stratum radiatum and oriens. Immunofluorescence signals from blood vessels and distinguishable glial cells were excluded from the quantification. To determine the synaptic localization of gephyrin, neuroligin-2, and β1 integrin, the vesicular GABA transporter (vGAT) signal was used as a presynaptic marker of inhibitory synapses. The analyzed puncta were classified as synaptic if they overlapped with vGAT-positive regions, which were defined by enlarging the vGAT mask by two pixels in all directions. The areas of gephyrin and neuroligin-2 clusters in vGAT-positive inhibitory synapses were analyzed using the particle Analysis function in ImageJ. Puncta density was calculated as the number of puncta divided by the analyzed area.

### Compounds and chemicals

All chemicals were purchased from Sigma-Aldrich, unless otherwise specified. Tetrodotoxin (TTX) was obtained from Latoxan, and 6,7-dinitroquinoxaline-2,3-dione (DNQX) from Tocris Bioscience. The neurolide-2 peptide and scrambled control were synthesized on demand by Schafer-N. Neurolide-2 consists of a lysine backbone conjugated with four HSEGLFQRA peptides, representing a specific sequence of neuroligin-2 that is critical for its interaction with neurexins. The scrambled control peptide was constructed on the same lysine backbone but contained four FEASHRLQG peptides derived from a shuffled version of the original neurolide-2 sequence. By mimicking the neuroligin-2 binding site, neurolide-2 acts as a competitive inhibitor, effectively blocking the formation of new neuroligin-2–neurexin interactions. Both peptides were dissolved directly in aCSF. Neurolide-2 (50 μM) or its scrambled control peptide was bath applied for 12 min before NMDA application or high-frequency stimulation and remained present throughout the entire recording thereafter. In control experiments, neurolide-2 was applied alone for up to 40 min to assess its effect on baseline synaptic transmission. The selected concentration was based on previous reports demonstrating the effective disruption of Nlgn2–neurexin binding in neuronal preparations and in vivo (Van Der Kooij et al., 2014; Kim et al., 2016).

### Experimental design and statistical analysis

Our methodology followed several, predetermined guidelines. We intentionally retained all the collected data points without removing the outliers. Experimental groups were assigned randomly, although the researchers were aware of the treatment conditions during both the data collection and analysis phases. Sample sizes were determined through power calculations prior to the experiments, with the goal of achieving 0.80 statistical power or greater. All statistical analyses were performed using GraphPad Prism 8. Each experimental group contained data from independent experiments using tissue samples obtained from a minimum of three animals of both sexes. We evaluated data distributions for normality using the Shapiro–Wilk test. For two-group comparisons, we selected appropriate tests based on distribution characteristics using Student's *t* test (paired or unpaired) for parametric data and Mann–Whitney *U* or Wilcoxon test for nonparametric data. Multigroup comparisons were performed using one-way or two-way analysis of variance (ANOVA), followed by Tukey's or Sidak's post hoc test when appropriate. We used standard notation to indicate statistical significance: ns, *p* > 0.05; *p ≤ 0.05; **p ≤ 0.01; ***p ≤ 0.001. Complete experimental details, including specific sample sizes and statistical methods, are provided in the corresponding figure captions. All values are shown as mean ± SEM.

## Results

### Interference with neuroligin-2–neurexin adhesion impairs NMDA-induced iLTP

To investigate how synaptic adhesion shapes GABAergic plasticity, we focused on the interaction between neuroligin-2 (Nlgn2) and presynaptic neurexins, which organizes inhibitory synapse structure and function. We used neurolide-2, a synthetic peptide (HSEGLFQRA) that mimics a conserved Nlgn2 motif required for neurexin-1β binding, to competitively block the formation of new Nlgn2–neurexin complexes. The peptide was synthesized as a tetramer to increase avidity by using a lysine backbone. Its action and specificity have been validated in previous studies (Van Der Kooij et al., 2014; Kim et al., 2016). To assess the physiological consequences of interference with Nlgn2-dependent adhesion, we recorded miniature inhibitory postsynaptic currents (mIPSCs) in CA1 pyramidal neurons in acute hippocampal slices. Neurolide-2 (50 µM) was bath applied, whereas mIPSCs were pharmacologically isolated using DNQX (20 µM) and tetrodotoxin (1 µM; Fig. 1*A*). Under baseline conditions, neurolide-2 did not alter mIPSC amplitude or frequency, indicating that inhibitory transmission was unaffected acutely (Fig. S1). However, when we induced iLTP using NMDA (20 µM, 3 min), a clear difference emerged (Fig. 1*B*). In control slices treated with scrambled peptide, NMDA triggered a stable increase in the mIPSC amplitude (Fig. 1*C*,*D*), consistent with earlier reports of GABAergic iLTP (Wiera et al., 2021). In contrast, neurolide-2 completely prevented this potentiation (Fig. 1*C*,*D*). Paired comparisons within individual cells confirmed a reliable iLTP in controls and a failure of potentiation in the neurolide-2 group (Fig. 1*E*). In line with the postsynaptic locus of expression (Petrini et al., 2014), mIPSC frequency remained unchanged following NMDA application in either condition (Fig. S2). Collectively, these results show that Nlgn2-dependent adhesion is required for the expression of NMDA-induced iLTP at inhibitory synapses in CA1 pyramidal neurons.

### Neuroligin-2–neurexin adhesion is required for gephyrin recruitment at inhibitory synapses during iLTP

To test whether the impairment of iLTP in neurolide-2-treated slices reflects alterations in postsynaptic structure, we examined gephyrin, the principal scaffolding protein at inhibitory synapses. Earlier work showed that NMDA-induced iLTP in hippocampal slices is accompanied by an increase in the size of synaptic gephyrin clusters (Petrini et al., 2014; Wiera et al., 2021, 2022). Using confocal imaging of hippocampal sections for gephyrin and vGAT, we quantified the area of gephyrin clusters within the vGAT-positive inhibitory synapses in the CA1 region. Four experimental groups were analyzed: control and NMDA-treated slices, each incubated with either scrambled control peptide or neurolide-2. In sham-treated slices, NMDA application caused a clear enlargement of gephyrin synaptic clusters (Fig. 2*A*), which was evident in both stratum radiatum (Fig. 2*B*) and stratum oriens (Fig. 2*C*). This confirms that iLTP induction is associated with structural remodeling of inhibitory synapses. In contrast, NMDA-treated slices exposed to neurolide-2 showed no detectable changes in gephyrin cluster area (Fig. 2*A–C*). Their labeling pattern closely matched that of sham controls, indicating that structural plasticity was suppressed. We also noted that neurolide-2 alone reduced baseline gephyrin cluster area at vGAT-positive inhibitory synapses in the stratum oriens, while synapses in stratum radiatum were unaffected (Fig. 2*A–C*). Neither iLTP induction nor neurolide-2 treatment altered the average fluorescence intensity of synaptic gephyrin (Fig. S3*A*,*B*). Taken together, these observations show that NMDA-induced iLTP in CA1 promotes gephyrin accumulation at inhibitory synapses across both apical and basal dendritic compartments. This form of structural remodeling depends on intact neurexin–neuroligin-2 adhesion, as interfering with this interaction blocks gephyrin recruitment and prevents the expression of iLTP.

### Neuroligin-2 recruitment to inhibitory synapses during NMDA-iLTP is layer-specific and depends on neurexin binding

To examine whether neuroligin-2 is recruited to inhibitory synapses during NMDA-induced iLTP, and whether this process requires neurexin binding, we performed immunostaining for neuroligin-2 and vGAT in the CA1 under four conditions: scrambled peptide control, neurolide-2 alone, NMDA-induced iLTP, and NMDA-induced iLTP in the presence of neurolide-2 (Fig. 3*A*). Both in stratum oriens and radiatum, synaptic neuroligin-2 clusters in control slices and neurolide-2–only slices were similar in size and distribution (radiatum: *p* = 0.51; oriens: *p* = 0.99), showing that neurolide-2 by itself does not alter neuroligin-2 localization. After induction of NMDA-iLTP, neuroligin-2 cluster area increased markedly, but this effect was restricted to stratum oriens (control vs iLTP; radiatum: *p* = 0.92; oriens: *p* = 2 × 10^−5^; Fig. 3*B*,*C*). This increase was absent when neurolide-2 was present before, during, and after NMDA treatment. In both layers, neuroligin-2 cluster size was significantly smaller in iLTP slices exposed to neurolide-2 compared with the iLTP group without the peptide (radiatum: *p* = 0.018; oriens: *p* = 6 × 10^−6^; Fig. 3*B*,*C*). The average fluorescence intensity of synaptic neuroligin-2 remained unchanged following either iLTP induction or neurolide-2 treatment (Fig. S3*C*,*D*). These results show that neuroligin-2 is recruited to inhibitory synapses in the basal dendrites (stratum oriens) during NMDA-induced iLTP and that this process depends on intact neurexin binding. In contrast, synapses in the apical dendrites (stratum radiatum) did not exhibit neuroligin-2 accumulation following NMDA stimulation. Notably, neurolide-2 treatment during iLTP induction reduced the neuroligin-2 area at vGAT-positive synapses in the stratum radiatum, suggesting a suppressive effect in this layer.

Recent studies have shown that NMDA-induced iLTP in CA1 pyramidal neurons depends on another group of adhesion proteins, integrins, which anchor the cytoskeleton to the extracellular matrix. Among brain-expressed integrins, those containing the β_1_ subunit are activated during iLTP induction, and blocking this activation disrupts GABAergic plasticity (Wiera et al., 2022). Under control conditions, NMDA-induced iLTP led to an increase in the fraction of vGAT-positive inhibitory synapses colocalized with the active extended form of integrin β_1_ in both the stratum oriens and stratum radiatum. However, when iLTP was impaired by neurolide-2, this effect was abolished (Fig. S4). These findings suggest that the reorganization of neuroligin-2-dependent adhesion precedes and may be required for the activation of β_1_ integrins during GABAergic iLTP.

### The expression of NMDA-iLTP requires neuroligin-2–neurexin adhesion within a brief post-induction window

To determine how the timing of interference with neurexin–neuroligin-2 complex formation affects GABAergic plasticity, we examined changes in mIPSC amplitude in CA1 pyramidal neurons following NMDA-induced iLTP while applying neurolide-2 at distinct time points. Neurolide-2 was applied under four different conditions: (1) during NMDA application (0–3’), (2) starting after completion of NMDA application (from 3’), (3) starting 10 min after induction, or (4) starting 15 min after induction. When neurolide-2 was applied during NMDA exposure (0–3’), iLTP was fully expressed (117 ± 5% of baseline; Fig. 4*A*), as shown by a significant and sustained increase in mIPSC amplitude (Fig. 4*B*,*C*). However, when neurolide-2 was applied during various time windows within post-induction period (3’–30’), the effect on iLTP was strongly diversified, indicating that within this time period, the iLTP consolidation process is critically sensitive to adhesion interactions between neuroligin-2 and neurexin. Indeed, when neurolide-2 was applied immediately after NMDA treatment, not only iLTP was disrupted, but mIPSC amplitudes were also reduced below baseline levels, indicative of iLTD (90 ± 3%; Fig. 4*A–C*). In the case of neurolide-2 application from 10 min after iLTP induction, there was an initial trend to build up iLTP as long as neurolide-2 was absent, but when this peptide was applied (at 10’), mIPSC amplitude relaxed to the value observed during baseline recording, revealing iLTP disruption (99 ± 1%). Interestingly, when neurolide-2 was applied 15 min after NMDA administration (from 15’), iLTP was unaffected and remained robust, with a magnitude comparable to that in the sham treatment condition (113 ± 3%; Fig. 4*A–C*). These results indicate that synaptic recruitment and stabilization of neuroligin-2 likely occur within the first 10 min following iLTP induction. Interfering with neurexin binding during this window is sufficient to disrupt the development of plasticity, whereas neurolide-2 application at later time points does not reverse established iLTP. Together, these findings define a critical postinduction time window during which the formation of new neuroligin-2–neurexin interactions is necessary to consolidate iLTP at inhibitory synapses.

### Disruption of neuroligin-2–neurexin interaction prevents iLTP at dendritic and perisomatic inhibitory inputs

Previous studies have shown that neuroligin-2 exhibits clear synapse-specific localization and functions (Gibson et al., 2009; Horn and Nicoll, 2018; Groisman et al., 2023). To test whether interference with the formation of new neurexin–neuroligin-2 complexes with neurolide-2 affects iLTP at defined inhibitory inputs, we monitored optogenetically evoked IPSCs from somatostatin (SST)- or parvalbumin (PV)-expressing interneurons in CA1 pyramidal cells (Fig. 5*A*,*E*). As expected, these IPSCs differed strongly in their rise times (*p* = 10^−9^), consistent with the electrotonic filtering of dendritic SST inputs compared with the faster perisomatic PV synapses (Fig. S5).

A brief NMDA exposure (1 min 45 s) produced a stable iLTP of SST → PC IPSCs in sham-treated slices, reaching 119 ± 7.2% of the baseline (Fig. 5*B*,*C*), consistent with earlier observations (Wiera et al., 2024). In contrast, continuous presence of neurolide-2 prevented this increase, and SST → PC IPSCs remained close to baseline (89.7 ± 6.5%; Fig. 5*B–D*). The NMDA protocol did not alter IPSC kinetics (Fig. S6).

We next assessed whether this effect extends to perisomatic inhibition. In slices expressing channelrhodopsin-2 in PV-positive interneurons, NMDA application for 1 min 15 s induced robust iLTP of PV → PC IPSCs (135 ± 7.5% of baseline; Fig. 5*E–G*). This effect was significantly reduced in the presence of neurolide-2, with IPSCs reaching only 110 ± 5.2% of baseline (Fig. 5*G*,*H*). Across both input types, IPSC amplitudes after NMDA remained statistically unchanged relative to preinduction values when neurolide-2 was present, whereas sham-treated slices showed reliable potentiation (Fig. 5*D*,*H*). Importantly, neither neurolide-2 nor its scrambled version altered baseline IPSC amplitudes at SST → PC or PV → PC synapses (Fig. 6*A–C*). These findings indicate that neurolide-2 disrupts the maintenance of iLTP at both dendritic SST-mediated and perisomatic PV-mediated inhibitory synapses.

### Neuroligin-2 is required for consolidation of heterosynaptic iLTP at SST inputs to CA1 pyramidal neurons

Numerous forms of GABAergic plasticity show heterosynaptic induction, as they are triggered by activity at neighboring excitatory synapses rather than by direct activation of the inhibitory input itself (Chevaleyre and Castillo, 2003; Field et al., 2020; Ravasenga et al., 2022). While NMDA application has been useful for revealing mechanisms of iLTP, it lacks physiological specificity and may not fully reflect how GABAergic plasticity is engaged in functioning networks. To address this issue, we tested whether a more physiologically relevant protocol, high-frequency stimulation (HFS) of the CA3 → CA1 excitatory pathway paired with postsynaptic depolarization, could induce heterosynaptic iLTP at identified inhibitory inputs.

**Figure 7.**
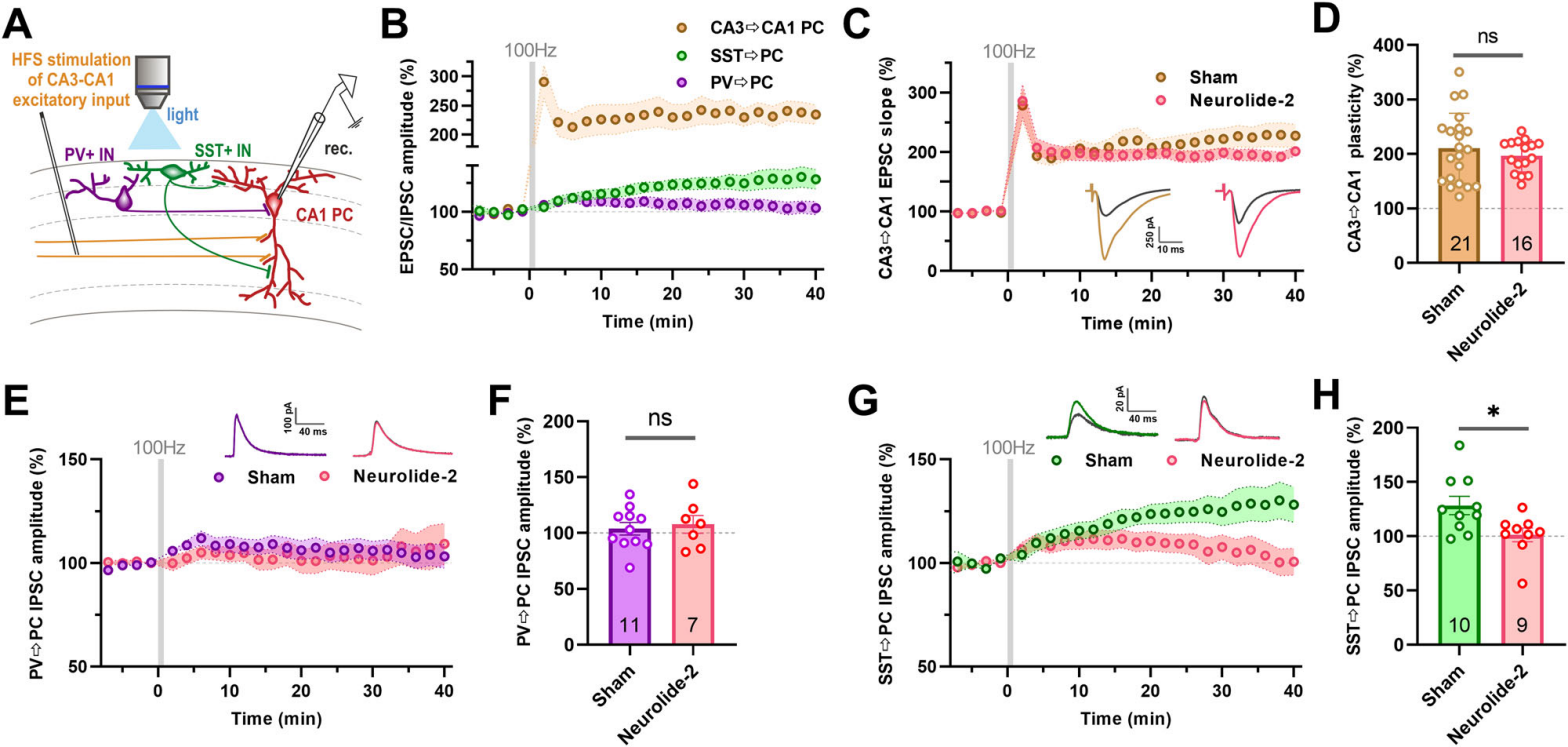
High-frequency stimulation of CA3 → CA1 excitatory input induces heterosynaptic iLTP at SST → PC synapses in a neuroligin-2-dependent manner. ***A***, Experimental configuration. To induce excitatory LTP and heterosynaptic iLTP, CA3 Schaffer collaterals (orange) were stimulated at 100 Hz (HFS, high-frequency stimulation), while CA1 pyramidal cells (PCs) were held at 0 mV using patch-clamp electrode (rec.). Optogenetic stimulation of SST (green) or PV (magenta) interneurons with blue light (2 ms) and whole-cell recordings of IPSCs (−60 mV holding potential) and CA3 → CA1 EPSCs (−70 mV holding potential) were performed concurrently in the same postsynaptic neuron. ***B***, Time course of normalized PSCs amplitudes recorded in CA1 pyramidal cells in response to electrical stimulation of CA3 → CA1 excitatory input (EPSC recorded at −70 mV) and optogenetically evoked SST → PC or PV → PC IPSC (recorded at −60 mV) in control slices. The high-frequency 100 Hz stimulation (gray bar) elicited robust excitatory LTP and heterosynaptic SST → PC iLTP, but not PV → PC plasticity. Shaded areas represent SEM. Mean and individual values of synaptic plasticity in the control group across all analyzed excitatory and inhibitory inputs to CA1 pyramidal cells are shown in panels ***D***, ***F***, and ***H***. ***C***, ***D***, CA3 → CA1 excitatory LTP is unaffected by neurolide-2 (219 ± 12% vs 196.9 ± 7.4%; *t*_(35)_ = 0.79, *p* = 0.43). Representative traces from individual experiments show EPSCs before (gray) and 36–40 min after HFS (colored). ***E–H***, Effect of neurolide-2 on GABAergic heterosynaptic plasticity in SST → PC (***E***, ***F***) and PV → PC (***G***, ***H***) pathways. A two-way ANOVA revealed a significant main effect of synapse type (*F*_(1,33)_ = 4.64, *p* = 0.038). Sidak's post hoc tests showed adjusted *p* = 0.020 for SST → PC synapses and *p* = 0.92 for PV → PC synapses, confirming that the peptide effect was specific to SST → PC connections. Representative traces show optogenetically evoked IPSCs before (gray) and 35–40 min after HFS (colored). Error bar graphs present mean ± SEM values measured 36–40 min post-HFS; numbers within bars indicate the number of recorded cells. *P* < 0.05; ns, not significant; unpaired *t* test.

We applied three trains of 100 Hz stimulation (1 s each, spaced 20 s apart) to Schaffer collaterals while holding CA1 pyramidal neuron at 0 mV, a protocol known to reliably induce LTP at excitatory synapses (Jiang et al., 2017). To assess inhibitory plasticity, we optogenetically stimulated either SST- or PV-expressing interneurons and recorded evoked IPSCs in CA1 pyramidal neurons before and after HFS (Fig. 7*A*). As expected, excitatory CA3 → CA1 synapses exhibited robust LTP, with EPSC amplitudes increasing to 219 ± 12% of baseline (*n* = 21; Fig. 7*B*). Importantly, the same protocol led to a sustained enhancement of SST → PC IPSCs, reaching 138 ± 8% of baseline (*n* = 10; Fig. 7*B–D*), without detectable changes in IPSC kinetics or short-term plasticity (Fig. S7). In contrast, PV → PC IPSC amplitudes remained unchanged (104 ± 5%; *n* = 11; Fig. 7*B*), and neither IPSC kinetic properties nor short-term plasticity were affected (Fig. S8), confirming the synapse-specific nature of HFS-induced inhibitory plasticity.

To determine whether neurexin–neuroligin-2 interactions are necessary for this form of heterosynaptic iLTP, we have repeated the same experimental protocols in the presence of neurolide-2. Excitatory LTP at CA3 → CA1 synapses remained intact in the presence of neurolide-2 (196.9 ± 7.4% of baseline; *p* = 0.43 vs sham; Fig. 7*C*,*D*), confirming that neurolide-2 does not interfere with excitatory synapses. PV → PC responses also remained unaffected (108 ± 8%; *p* = 0.68 vs sham; Fig. 7*E*,*F*). In contrast, potentiation at SST → PC synapses was abolished, with IPSC amplitudes remaining at 101 ± 6% of baseline (*p* = 0.02 vs sham; Fig. 7*G*,*H*). Notably, in the neurolide-2 group, SST → PC IPSCs initially exhibited potentiation but began to decline ∼20 min after HFS, gradually returning to baseline levels. This temporal profile suggests that while the initial expression of SST → PC iLTP is intact, its maintenance requires neuroligin-2-mediated transsynaptic adhesion.

## Discussion

In this study, we sought to determine the role of neuroligin-2 (Nlgn2) in the long-term plasticity of GABAergic synapses in hippocampal CA1 pyramidal neurons. Using neurolide-2, a synthetic multivalent peptide that selectively interferes with Nlgn2–neurexin binding, we show that perisynaptic and transsynaptic adhesion between Nlgn2 and neurexins is required for the expression and maintenance of NMDA-induced iLTP. Impairment of this adhesion prevented iLTP at both dendritic and perisomatic inhibitory synapses (Figs. 1, 5) and blocked the activity-driven accumulation of postsynaptic gephyrin (Figs. 2, 3). Importantly, neurolide-2 effectively abolished iLTP when applied within a restricted time window (ca. 10 min) from plasticity induction, identifying thus a time interval during which new interactions between Nlgn2 and neurexins are necessary to stabilize plastic synaptic changes (Fig. 4). Finally, we demonstrate that a physiologically relevant plasticity paradigm, a high-frequency stimulation of Schaffer collateral excitatory input to CA1 pyramidal cells, induces heterosynaptic iLTP at SST → PC synapses (but not at PV → PC) and that the maintenance of this new form of GABAergic plasticity also depends on Nlgn2 (Fig. 7). Together, these results identify neuroligin-2 as a key synaptic determinant required for early maintenance of iLTP at inhibitory synapses in CA1.

### Neuroligin-2–neurexin adhesion supports consolidation of inhibitory plasticity

Our findings locate Nlgn2–neurexin adhesion at the core of mechanisms that stabilize long-term changes in GABAergic synaptic strength. Importantly, we show that selective disruption of Nlgn2–neurexin binding prevents iLTP induced by two different protocols: transient NMDA administration and by physiologically patterned CA3 → CA1 high-frequency stimulation paired with PC depolarization. This observation indicates that Nlgn2–neurexin complex formation is indeed a key player in the inhibitory plasticity arising from different forms of network or cellular activity. It is noteworthy that, although neuroligin-2 is a postsynaptic transmembrane adhesion molecule whose extracellular domain binds neurexins, this interaction forms adhesion-based signaling platforms. These cascades can induce changes in translation or CaMKII-dependent phosphorylation of GABA_A_ receptor subunits and promote gephyrin scaffold expansion, processes that are central to inhibitory synaptic plasticity (Petrini et al., 2014; Flores et al., 2015; Rajgor et al., 2020). Consistent with our observation that Nlgn2 is essential for iLTP, recent evidence demonstrates that preventing phosphorylation of Nlgn2 at Ser714 stabilizes it in the synapse and enhances inhibitory transmission, highlighting a potential mechanism by which intracellular signaling cascades can regulate the adhesion during inhibitory plasticity (Halff et al., 2022).

We further found that interfering with Nlgn2–neurexin interaction not only blocked gephyrin accumulation at inhibitory synapses during iLTP but also induced synaptic weakening when the disruption occurs immediately after plasticity induction (Fig. 4). This indicates that the reorganization of Nlgn2-dependent adhesion provides early structural support that is critical for converting transient plasticity into stable GABAergic potentiation within time scale of our recordings. When this process is interrupted within the first 10 min post-induction, gephyrin fails to accumulate, iLTP is not maintained, and inhibitory synapses instead undergo depression. The analogous stabilization phase is well documented at excitatory synapses, where neuroligin-1–neurexin interactions facilitate AMPA receptor recruitment and dendritic spine growth during early LTP (Jiang et al., 2017; Wu et al., 2019). Similarly, integrin signaling, which is required for actin cytoskeleton stabilization and glutamate receptor anchoring, is essential during a restricted post-induction window to consolidate excitatory LTP (Kramár et al., 2006; Babayan et al., 2012). Importantly, integrins also contribute to inhibitory plasticity, as active β1 integrins become enriched at inhibitory synapses during NMDA-iLTP, and pharmacological blockade of β1 integrin activation prevents the induction of iLTP (Wiera et al., 2022). We found that this activation is impaired when Nlgn2–neurexin interactions are disrupted, suggesting that remodeling of Nlgn2-dependent adhesion precedes and possibly enables integrin β1 activation in inhibitory synapses.

**Figure 8.**
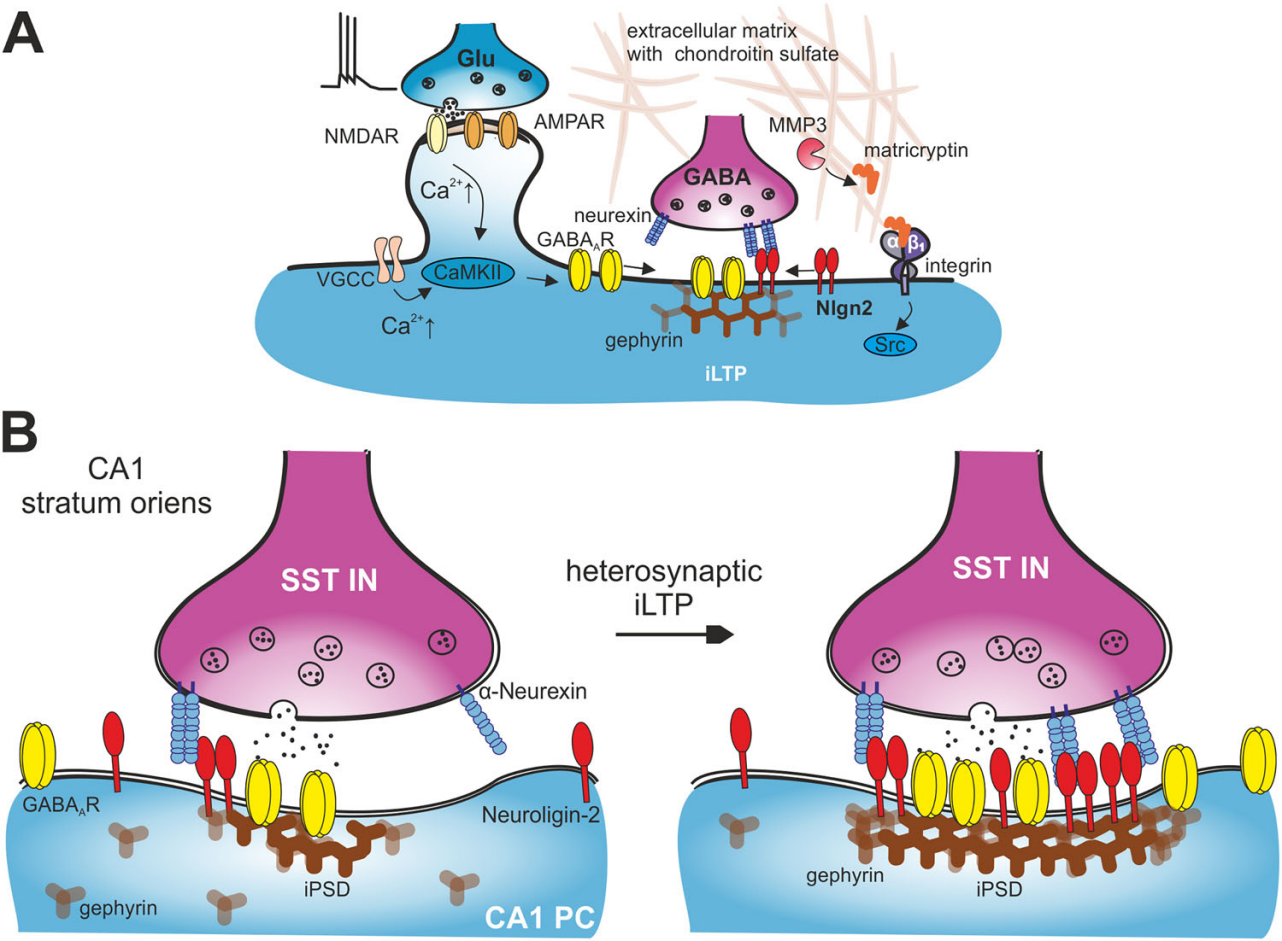
Conceptual model of neuroligin-2-dependent inhibitory synaptic plasticity in hippocampal CA1. ***A***, Molecular mechanisms of iLTP at GABAergic synapses. Postsynaptic Ca^2+^ influx, mediated by NMDA receptors and voltage-gated Ca^2+^ channels, activates CaMKII signaling, which promotes the stabilization and synaptic retention of GABA_A_ receptors via clustering with gephyrin scaffold. Concurrently, Matrix Metalloproteinase-3 (MMP-3) cleaves extracellular matrix substrates (such as chondroitin sulfate proteoglycans), thereby activating β1-integrin-containing complexes. This cascade modulates synaptic adhesion and receptor confinement to induce iLTP. Neuroligin-2, via interactions with presynaptic partners such as neurexins, orchestrates the reorganization of the synaptic adhesion apparatus and consolidates the plastic change. ***B***, Model of heterosynaptic iLTP at somatostatin-expressing interneuron (SST IN) inputs onto CA1 pyramidal cells (CA1 PC) in the stratum oriens with emphasis on involvement of Neuroligin2-neurexin complexes. Potentiation involves the increased postsynaptic accumulation of neuroligin-2, gephyrin, and GABA_A_ receptors at inhibitory postsynaptic densities (iPSD), independent of presynaptic modifications. This framework establishes neuroligin-2 as a central organizer coupling extracellular cues to postsynaptic scaffold remodeling during the consolidation of inhibitory synaptic strengthening.

Neurolide-2 targets the neurexin-binding site of Nlgn2 (Van der Kooij et al., 2014; Kim et al., 2016). It is unlikely that neurolide-2 directly affects other extracellular or adhesion proteins such as β1-integrins or extracellular proteases, which are also implicated in GABAergic plasticity (Wiera et al., 2021, 2022). Consistent with this, neurolide-2 did not alter baseline inhibition or gephyrin clustering, which are highly sensitive to interference with integrin functioning or MMP3 activity. Together with earlier findings on the extracellular matrix and integrins in GABAergic plasticity (Wiera et al., 2021, 2022; Jabłońska et al., 2024), the present results support a model in which NMDA receptor activation initiates processes leading to extracellular matrix remodeling and integrin signaling, which is paralleled by synaptic recruitment and stabilization of gephyrin scaffolds and neuroligin-2 in GABAergic postsynaptic density required for the consolidation of inhibitory strength (Wiera and Mozrzymas, 2021). This conceptual framework is illustrated in Figure 8.

Finally, NMDA-dependent iLTP involves rapid reorganization of gephyrin nanoclusters (Pennacchietti et al., 2017), and recent studies have shown that proteolytic cleavage of Nlgn2 by matrix metalloproteinases (MMPs) leads to dispersion of GABA_A_ receptors from synaptic structures and weakened inhibition (Xu et al., 2024). Interestingly, MMP3 is critically involved in NMDA-induced iLTP (Wiera et al., 2021), suggesting that proteolytic remodeling of GABAergic adhesome may play a key role in inhibitory plasticity. Together, the results presented here support a model in which the formation of Nlgn2–neurexin complexes serves as adhesion platforms that are required not only for the maintenance of inhibitory potentiation but can also contribute to synaptic weakening when dismantled in early phases of plasticity induction. This positions Nlgn2 as a molecular checkpoint linking extracellular adhesion to intracellular scaffold remodeling during bidirectional GABAergic plasticity.

### Neuroligin-2 and input specificity of GABAergic plasticity

The interplay between inhibition and excitation depends not only on the strength of individual synapses but also on the spatial and temporal coordination of coplasticity at excitatory and inhibitory inputs (Agnes and Vogels, 2024). Inhibitory synapses exhibit diverse forms of long-term plasticity that can be induced via homosynaptic or heterosynaptic mechanisms. In the latter, the duration of NMDA receptor activation determines the direction of GABAergic plasticity (Muir et al., 2010; Petrini et al., 2014; Barberis, 2020), along with concurrent excitatory plasticity (Field et al., 2020; Wiera et al., 2024). Interestingly, this coplasticity can follow a different pattern when the postsynaptic cell is an interneuron (Brzdąk et al., 2023; Jabłońska et al., 2025). The bidirectional coplasticity of excitatory and inhibitory drive onto the same postsynaptic pyramidal neuron represents a local mechanism by which the excitatory/inhibitory (E/I) ratio is dynamically adjusted in response to activity (Ravasenga et al., 2022). Our findings add that Nlgn2–neurexin adhesion is a critical structural checkpoint in this heterosynaptic process, required to stabilize GABAergic plasticity, thereby enabling lasting shifts in the local E/I ratio. This, in turn, suggests a mechanism by which dysfunctions in Nlgn2-dependent adhesion may disrupt neuronal E/I balance in neurological disorders associated with neuroligins (Liang et al., 2015; Krueger-Burg et al., 2017).

Owing to the diversity of interneurons, the forms and mechanisms of GABAergic plasticity exhibit remarkable input specificity. In the cortex, NMDA preferentially strengthens dendritic synapses formed by SST-positive interneurons, whereas perisomatic synapses formed by PV-positive interneurons are not equally potentiated (Chiu et al., 2018). In hippocampal CA1 pyramidal cells, both SST → PC and PV → PC synapses can exhibit iLTP, but with distinct induction requirements: SST synapses require longer NMDA exposure for iLTP induction compared with PV synapse (Wiera et al., 2024). This specificity may reflect differences in GABA_A_ receptor subunit composition (Szodorai et al., 2018), the presence of auxiliary proteins such as LHFPL4 and Shisa7 (Han et al., 2021; Wu et al., 2022b), or distinct adhesion molecules, including neuroligin-3 (Horn and Nicoll, 2018) and dystroglycan (Pribiag et al., 2014; Früh et al., 2016). Our findings provide a new perspective on the synapse specificity of GABAergic plasticity by demonstrating that iLTP at both SST → PC and PV → PC synapses depends on Nlgn2 when potentiation is induced by NMDA receptor activation. Suggesting that the role played by Nlgn2–neurexin adhesion in inhibitory plasticity is not input-specific but it functions as unifying mechanism permitting GABAergic plasticity at distinct types of GABAergic inputs. This appears to contrast with previous studies reporting that constitutive Nlgn2 knock-out reduced inhibitory input from PV but not SST interneurons (Gibson et al., 2009; Poulopoulos et al., 2009; Jedlicka et al., 2011). These studies focused on developmental assembly and basal function of inhibitory synapses, whereas the present work examined adult hippocampal networks and activity-dependent plasticity, with Nlgn2–neurexin interactions disrupted acutely and reversibly by neurolide-2 rather than by global knock-out. These observations therefore suggest that developmental formation and maintenance of inhibitory synapses and adult activity-dependent plasticity likely depend on distinct molecular processes involving the Nlgn2–neurexin system.

In the present study, we describe novel physiological stimuli that induce synapse-specific heterosynaptic GABAergic plasticity. High-frequency stimulation applied to Schaffer collaterals, together with depolarization of the postsynaptic pyramidal neuron to 0 mV, resulted in SST → PC iLTP, which occurred alongside CA3 → CA1 excitatory LTP. Interestingly, this protocol has not evoked concurrent plasticity at PV → PC synapses. Importantly, this heterosynaptic SST → PC iLTP remained dependent on Nlgn2, although only for maintenance and not for the first phase of induction (Fig. 7). Similarly, MDGA1a protein interacting with Nlgn2 selectively regulates GABAergic synapses on distal dendrites, with no effect on proximal dendrites or soma (Zeppillo et al., 2024). Overall, our findings indicate that dendritic inhibitory synapses can monitor local excitatory activity and respond with long-term GABAergic plasticity. Such a mechanism acts to preserve the local dendritic E/I equilibrium within space and time, with Nlgn2 required for the consolidation of iLTP but not excitatory plasticity.

### Conclusion

Dysregulation of the E/I balance is a common denominator in numerous neurodevelopmental and psychiatric disorders, including autism spectrum disorder, schizophrenia, and epilepsy (Tang et al., 2021). Genetic variants of neuroligins, neurexins, and MDGAs have been implicated in these conditions (Krueger-Burg et al., 2017; Ali et al., 2020). However, the precise synaptic mechanisms linking these synapse organizers to network-level dysfunction remain poorly understood. Our study identifies a key function of Nlgn2 in consolidating activity-induced changes at GABAergic synapses. Impairment of this mechanism may contribute to the E/I imbalance observed in these disorders. In this light, it will be important to determine how specific pathogenic Nlgn2 mutations affect not only synapse formation but also the stabilization of inhibitory plasticity. Moreover, the temporally restricted role of Nlgn2 in inhibitory plasticity suggests that the precise timing of interference with Nlgn2–neurexin adhesion may offer a strategy to recalibrate dysfunctional inhibitory circuits.

While neurolide-2 is a well-characterized and selective tool for transiently disrupting the formation of Nlgn2–neurexin complexes (Van Der Kooij et al., 2014; Kim et al., 2016), our present conclusions are based on this single mode of interference. Future work combining neuron-specific conditional Nlgn2 deletion or viral CRISPR/Cas9 knockdown with pathway-defined electrophysiology will be essential to further scrutinize the causal role of Nlgn2 in GABAergic plasticity in the mature brain.

Astrocytes have recently been shown to express Nlgn2 and neurexin isoforms at levels comparable to or even exceeding those of neurons and to form perisynaptic adhesion complexes governing synapse structure (Stogsdill et al., 2017; Ackerman et al., 2021; Tan and Eroglu, 2021). Our discovery that neuronal iLTP is Nlgn2–neurexin adhesion dependent opens the intriguing possibility that astrocyte-derived Nlgn2 scaffolds similarly contribute to the stabilization of GABAergic plasticity consolidation, linking glial adhesion dynamics to the stabilization of plastic changes in tetrapartite inhibitory synapses.

Our findings identify Nlgn2–neurexin interactions as a key molecular player that supports the consolidation of inhibitory synaptic potentiation within specific hippocampal pathways. The data suggest that Nlgn2-dependent adhesion is preferentially engaged by defined patterns of excitatory activity. This could represent an adhesion-based code for determining long-term plasticity eligibility at inhibitory inputs. This mechanism conceptually extends the framework of synaptic tagging and capture (Redondo and Morris, 2011) to include the inhibitory synapses.

## Data Availability

All data reported in this paper will be shared by the lead contact upon request.
